# 2-(4-Methyl­phen­yl)quinoline-4-carb­oxy­lic acid

**DOI:** 10.1107/S160053681203797X

**Published:** 2012-09-08

**Authors:** Raed A. Al-Qawasmeh, Monther A. Khanfar, Musa H. Abu Zarga, Murad A. AlDamen

**Affiliations:** a Department of Chemistry, The University of Jordan, Amman 11942, Jordan

## Abstract

In the title compound, C_17_H_13_NO_2_, the dihedral angle between the plane of the carb­oxy group and the quinoline mean plane is 45.05 (13)°, and that between the toluene ring mean plane and the quinoline mean plane is 25.29 (7)°. In the crystal, molecules are linked *via* O—H⋯.N hydrogen bonds, forming chains propagating along the *b*-axis direction. These chain are linked *via* C—H⋯O interactions, forming two-dimensional networks lying parallel to the *ab* plane.

## Related literature

For the importance of the quinoline carb­oxy­lic acid analogues in the synthesis of various compounds with pharmacological properties, see: Deady *et al.* (1999 ▶, 2011 ▶); Kalluraya & Sreenivasa (1998 ▶); Tseng *et al.* (2008 ▶); Kravchenko *et al.* (2005 ▶). The structure of the related compound 2-phenyl­quinoline-4-carboxlic acid is described by Blackburn *et al.* (1996 ▶). For a description of puckering analysis, see: Cremer & Pople (1975 ▶). For synthetic preparation, see: Pfitzinger (1886 ▶).
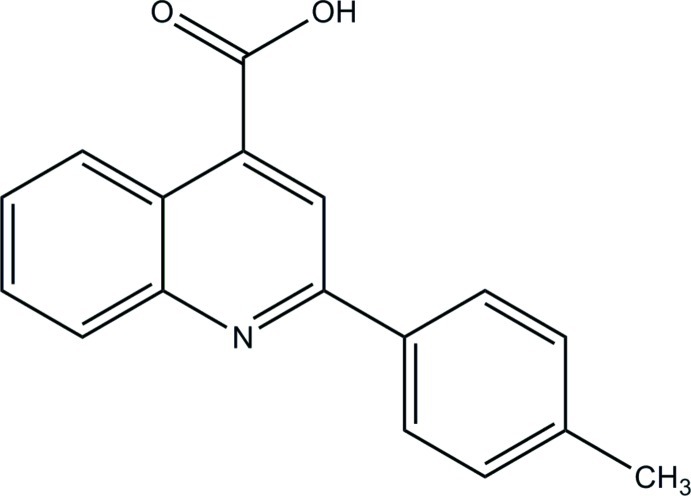


## Experimental

### 

#### Crystal data

C_17_H_13_NO_2_
*M* *_r_* = 263.28
Monoclinic, 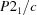

*a* = 4.1001 (6) Å
*b* = 15.3464 (11) Å
*c* = 20.3037 (17) Å
β = 90.859 (9)°
*V* = 1277.4 (2) Å^3^
*Z* = 4
Mo *K*α radiation
μ = 0.09 mm^−1^
*T* = 291 K
0.70 × 0.08 × 0.05 mm

#### Data collection

Oxford Diffraction Xcalibur Eos diffractometer
Absorption correction: analytical [*CrysAlis PRO* (Oxford Diffraction, 2009 ▶), based on expressions derived from Clark & Reid (1995 ▶)] *T*_min_ = 0.992, *T*_max_ = 0.999
4867 measured reflections
2238 independent reflections
1747 reflections with *I* > 2σ(*I*)
*R*_int_ = 0.025

#### Refinement

*R*[*F* ^2^ > 2σ(*F* ^2^)] = 0.049
*wR*(*F* ^2^) = 0.126
*S* = 1.04
2238 reflections
186 parameters
H atoms treated by a mixture of independent and constrained refinement
Δρ_max_ = 0.20 e Å^−3^
Δρ_min_ = −0.24 e Å^−3^

### 

Data collection: *CrysAlis PRO* (Oxford Diffraction, 2009 ▶); cell refinement: *CrysAlis PRO*; data reduction: *CrysAlis PRO*; program(s) used to solve structure: *SHELXS97* (Sheldrick, 2008 ▶); program(s) used to refine structure: *SHELXL97* (Sheldrick, 2008 ▶); molecular graphics: *OLEX2* (Dolomanov *et al.*, 2009 ▶); software used to prepare material for publication: *OLEX2*.

## Supplementary Material

Crystal structure: contains datablock(s) global, I. DOI: 10.1107/S160053681203797X/go2066sup1.cif

Structure factors: contains datablock(s) I. DOI: 10.1107/S160053681203797X/go2066Isup2.hkl

Supplementary material file. DOI: 10.1107/S160053681203797X/go2066Isup3.cml

Additional supplementary materials:  crystallographic information; 3D view; checkCIF report

## Figures and Tables

**Table 1:** Hydrogen-bond geometry (Å, °)

*D*—H⋯*A*	*D*—H	H⋯*A*	*D*⋯*A*	*D*—H⋯*A*
O1—H1⋯N1^i^	0.89 (3)	1.89 (3)	2.763 (2)	168 (2)
C3—H3⋯O1^ii^	0.93	2.51	3.233 (2)	135

Symmetry codes: (i) 
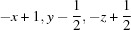
; (ii) 
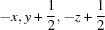
.

**Fig. 1.**
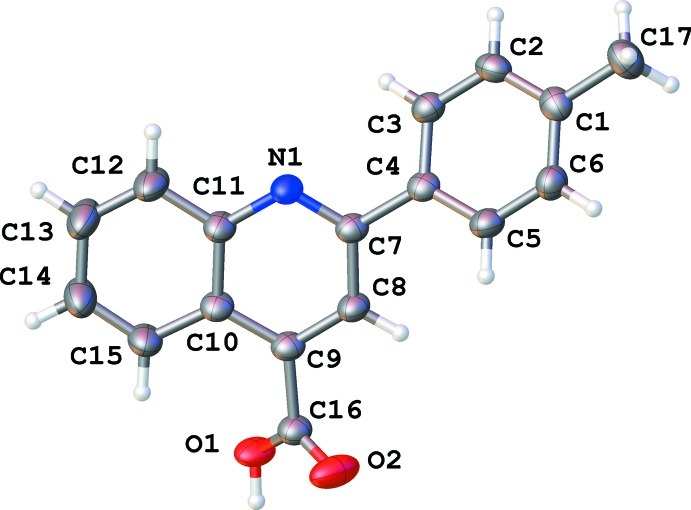
Molecular structure of the title compound. The thermal ellipsoids are drawn at the 30% probability level.

**Fig. 2.**
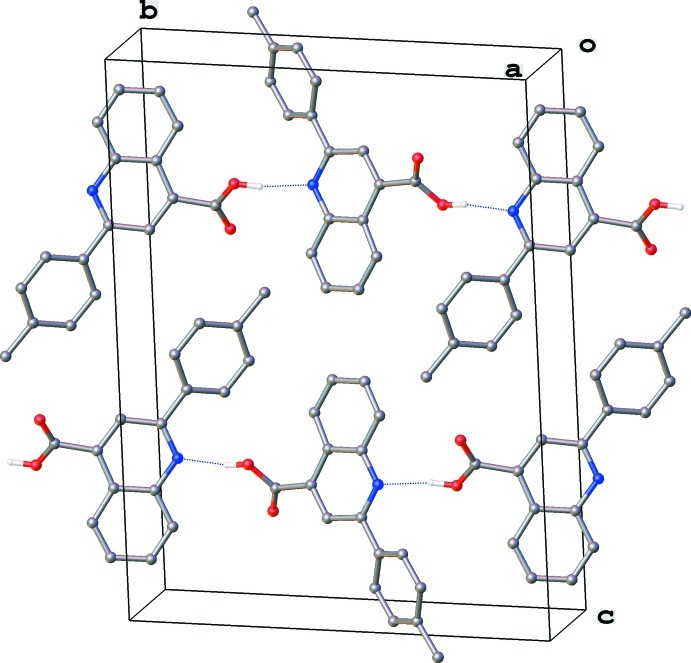
Packing diagram showing the one dimensional hydrogen bonded chains. Hydrogen atoms not involved in the hydrogen bonding are omitted for clarity.

## supplementary crystallographic information

### Comment

Quinoline derivatives are widely used as synthons for biologically important compounds (Tseng *et al.*, 2008), (Kravchenko *et al.*, 2005). In our group this moiety was used to synthesize new antitumor as well as antibacterial agents. The title molecule is shown in Fig. 1 with the numbering scheme. The dihedral angle between the plane of the carboxyl group and the quinoline mean plane is 45.05 (13)° and that between the toluene ring mean plane and the quinoline mean plane is 25.29 (7)°. The total puckering amplitude, *Q*, (Cremer & Pople, 1975) for the quinoline ring in the title compound is 0.044 (2)Å in the title compound as compared with the value of 0.080 (3) Å in the closely related compound 2-phenylquinoline-4-carboxlic acid (Blackburn *et al.*, 1996). There is a hydrogen bond between the carboxylic acid oxygen atom, O1 and N1 in the quinoline ring, Table 1, Figure 2. In addition the molecules are linked by a weak C-H..O interaction between C3 and O1, Table 1. There is π–π stacking between molecules Molecules are stacked above and below one another with unit translation along the a-axis so that rings containing N1 stack above those containing N1, the same applies to the rings containing C1 and C12. This results in π–π stacking between the molecules: i) between rings containing N1 (centroid Cg1) [Cg1···Cg1(-1+x, y, z), centroid to centroid distance: 4.1001 (13) Å, perpendicular distance between rings: 3.7681 (8) Å slippage: 1.616Å] and ii) between rings containing C1, (centroid Cg2), [Cg2···Cg2(-1+x ,y, z), centroid to centroid distance 4.1000 (14) Å, perpendicular distance between rings 3.3521 (8) Å, slippage 2.361Å] and iii) between rings containing C12 (centroid Cg3) Cg3···Cg3 (-1+x, y, z), [centroid to centroid) distance 4.1003 (17) Å, perpendicular distance between rings 3.7411 (11)Å, slippage 1.678Å].

### Experimental

The title compound was synthesized according to Pfitzinger reaction (Pfitzinger, 1886). Herein, we use the microwave technology for this synthesis, in a typical procedure: a mixture of isatin (1 mmole), acetophenone (1.05 equivalents) and potassium hydroxide (10 equivalents) in aqueous ethanol (10 ml) was placed in a closed vessel and irradiated with MW for 12 minutes at 140°C. The reaction mixture was acidified with acetic acid and the product was recrystallized from ethanol to produce white crystals with a melting point of 214–216 °C. Crystal with two different morphologies were found, cubic crystals which did not produce good diffraction and needle-shaped crystals. A large needle crystal was selected since the others were too small to provide good diffraction data.

### Refinement

All H atoms attached to C atoms were treated as riding atoms with C—H(aromatic), 0.93Å and C—H(methyl), 0.96Å, with *U*_iso_ = 1.2Ueq().

The H atom attached to the carboxylic -OH was located on a difference map and refined isotropically.

The positions of the methyl and hydroxyl hydrogen were checked on a final difference map.

### Crystal data

**Table:**

C_17_H_13_NO_2_	*F*(000) = 552
*M**_r_* = 263.28	*D*_x_ = 1.369 Mg m^−^^3^
Monoclinic, *P*2_1_/*c*	Mo *K*α radiation, λ = 0.71073 Å
Hall symbol: -P 2ybc	Cell parameters from 1144 reflections
*a* = 4.1001 (6) Å	θ = 3.0–29.0°
*b* = 15.3464 (11) Å	µ = 0.09 mm^−^^1^
*c* = 20.3037 (17) Å	*T* = 291 K
β = 90.859 (9)°	Needle, clear colourless
*V* = 1277.4 (2) Å^3^	0.70 × 0.08 × 0.05 mm
*Z* = 4	

### Data collection

**Table:**

Oxford Diffraction Xcalibur Eos diffractometer	2238 independent reflections
Radiation source: Enhance (Mo) X-ray Source	1747 reflections with *I* > 2σ(*I*)
Graphite monochromator	*R*_int_ = 0.025
Detector resolution: 16.0534 pixels mm^-1^	θ_max_ = 25.0°, θ_min_ = 3.3°
ω scans	*h* = −4→4
Absorption correction: analytical [*CrysAlis PRO* (Oxford Diffraction, 2009), based on expressions derived from Clark & Reid (1995)]	*k* = −18→12
*T*_min_ = 0.992, *T*_max_ = 0.999	*l* = −24→15
4867 measured reflections	

### Refinement

**Table:**

Refinement on *F*^2^	Primary atom site location: structure-invariant direct methods
Least-squares matrix: full	Secondary atom site location: difference Fourier map
*R*[*F*^2^ > 2σ(*F*^2^)] = 0.049	Hydrogen site location: inferred from neighbouring sites
*wR*(*F*^2^) = 0.126	H atoms treated by a mixture of independent and constrained refinement
*S* = 1.04	*w* = 1/[σ^2^(*F*_o_^2^) + (0.0574*P*)^2^ + 0.213*P*] where *P* = (*F*_o_^2^ + 2*F*_c_^2^)/3
2238 reflections	(Δ/σ)_max_ = 0.001
186 parameters	Δρ_max_ = 0.20 e Å^−^^3^
0 restraints	Δρ_min_ = −0.24 e Å^−^^3^

### Special details

**Table:**

Geometry. All e.s.d.'s (except the e.s.d. in the dihedral angle between two l.s. planes) are estimated using the full covariance matrix. The cell e.s.d.'s are taken into account individually in the estimation of e.s.d.'s in distances, angles and torsion angles; correlations between e.s.d.'s in cell parameters are only used when they are defined by crystal symmetry. An approximate (isotropic) treatment of cell e.s.d.'s is used for estimating e.s.d.'s involving l.s. planes.
Refinement. Refinement of *F*^2^ against ALL reflections. The weighted *R*-factor *wR* and goodness of fit *S* are based on *F*^2^, conventional *R*-factors *R* are based on *F*, with *F* set to zero for negative *F*^2^. The threshold expression of *F*^2^ > σ(*F*^2^) is used only for calculating *R*-factors(gt) *etc*. and is not relevant to the choice of reflections for refinement. *R*-factors based on *F*^2^ are statistically about twice as large as those based on *F*, and *R*- factors based on ALL data will be even larger.

### Fractional atomic coordinates and isotropic or equivalent isotropic displacement parameters (Å^2^)

**Table:**

	*x*	*y*	*z*	*U*_iso_*/*U*_eq_	
O1	0.5305 (4)	0.25205 (9)	0.25810 (7)	0.0455 (4)	
O2	0.7609 (5)	0.28629 (9)	0.16337 (8)	0.0636 (6)	
N1	0.3552 (4)	0.57466 (9)	0.24364 (7)	0.0347 (4)	
C1	−0.0757 (5)	0.69209 (12)	0.02784 (10)	0.0371 (5)	
C2	−0.1462 (5)	0.71004 (12)	0.09272 (10)	0.0385 (5)	
H2	−0.2786	0.7574	0.1024	0.046*	
C3	−0.0247 (5)	0.65926 (12)	0.14342 (9)	0.0350 (5)	
H3	−0.0740	0.6734	0.1867	0.042*	
C4	0.1703 (5)	0.58721 (11)	0.13094 (9)	0.0316 (5)	
C5	0.2352 (6)	0.56779 (12)	0.06552 (9)	0.0382 (5)	
H5	0.3618	0.5194	0.0556	0.046*	
C6	0.1145 (5)	0.61934 (13)	0.01517 (10)	0.0398 (5)	
H6	0.1615	0.6051	−0.0282	0.048*	
C7	0.3111 (5)	0.53556 (11)	0.18595 (9)	0.0315 (5)	
C8	0.3996 (5)	0.44729 (11)	0.17666 (9)	0.0346 (5)	
H8	0.3658	0.4214	0.1357	0.041*	
C9	0.5335 (5)	0.39967 (11)	0.22678 (9)	0.0331 (5)	
C10	0.5825 (5)	0.43954 (11)	0.28941 (9)	0.0346 (5)	
C11	0.4852 (6)	0.52799 (12)	0.29539 (9)	0.0365 (5)	
C12	0.5238 (7)	0.57039 (13)	0.35660 (10)	0.0529 (7)	
H12	0.4589	0.6281	0.3612	0.064*	
C13	0.6553 (8)	0.52727 (14)	0.40883 (11)	0.0630 (8)	
H13	0.6767	0.5555	0.4492	0.076*	
C14	0.7593 (7)	0.44087 (14)	0.40289 (11)	0.0585 (7)	
H14	0.8527	0.4126	0.4390	0.070*	
C15	0.7249 (6)	0.39806 (13)	0.34481 (10)	0.0456 (6)	
H15	0.7960	0.3407	0.3414	0.055*	
C16	0.6231 (5)	0.30697 (12)	0.21252 (10)	0.0360 (5)	
C17	−0.2037 (7)	0.74933 (14)	−0.02685 (11)	0.0532 (6)	
H17A	−0.2977	0.7137	−0.0610	0.064*	
H17B	−0.0278	0.7830	−0.0444	0.064*	
H17C	−0.3670	0.7878	−0.0100	0.064*	
H1	0.582 (7)	0.1965 (19)	0.2521 (12)	0.072 (8)*	

### Atomic displacement parameters (Å^2^)

**Table:**

	*U*^11^	*U*^22^	*U*^33^	*U*^12^	*U*^13^	*U*^23^
O1	0.0703 (12)	0.0208 (7)	0.0457 (9)	0.0014 (7)	0.0106 (8)	0.0032 (6)
O2	0.1040 (16)	0.0342 (8)	0.0534 (10)	0.0071 (9)	0.0301 (10)	−0.0010 (7)
N1	0.0480 (11)	0.0239 (8)	0.0324 (9)	−0.0006 (7)	0.0012 (8)	0.0016 (7)
C1	0.0379 (13)	0.0319 (11)	0.0414 (12)	−0.0059 (9)	−0.0056 (9)	0.0034 (9)
C2	0.0379 (13)	0.0281 (10)	0.0493 (13)	0.0039 (9)	−0.0034 (10)	0.0002 (9)
C3	0.0385 (13)	0.0307 (10)	0.0360 (11)	−0.0017 (9)	0.0016 (9)	−0.0031 (8)
C4	0.0364 (12)	0.0229 (9)	0.0356 (11)	−0.0058 (8)	0.0009 (9)	−0.0002 (8)
C5	0.0476 (14)	0.0283 (10)	0.0388 (12)	0.0018 (9)	0.0017 (10)	−0.0030 (8)
C6	0.0489 (14)	0.0389 (11)	0.0315 (11)	−0.0006 (10)	0.0002 (9)	0.0002 (9)
C7	0.0371 (12)	0.0237 (9)	0.0338 (11)	−0.0038 (8)	0.0043 (9)	0.0013 (8)
C8	0.0467 (13)	0.0237 (10)	0.0332 (11)	−0.0016 (9)	0.0003 (9)	−0.0018 (8)
C9	0.0396 (13)	0.0227 (9)	0.0369 (11)	−0.0029 (8)	0.0030 (9)	0.0010 (8)
C10	0.0440 (13)	0.0225 (9)	0.0371 (11)	−0.0047 (9)	0.0003 (9)	0.0021 (8)
C11	0.0520 (14)	0.0238 (10)	0.0338 (11)	−0.0029 (9)	−0.0004 (9)	0.0029 (8)
C12	0.090 (2)	0.0286 (11)	0.0396 (13)	0.0023 (12)	−0.0062 (12)	−0.0028 (9)
C13	0.110 (2)	0.0386 (13)	0.0395 (13)	−0.0017 (14)	−0.0164 (13)	−0.0051 (10)
C14	0.092 (2)	0.0380 (12)	0.0446 (14)	0.0000 (13)	−0.0220 (13)	0.0045 (10)
C15	0.0641 (16)	0.0280 (10)	0.0444 (13)	0.0025 (10)	−0.0095 (11)	0.0026 (9)
C16	0.0462 (14)	0.0246 (10)	0.0373 (11)	−0.0013 (9)	0.0015 (10)	0.0011 (8)
C17	0.0620 (17)	0.0482 (13)	0.0490 (13)	0.0059 (12)	−0.0083 (12)	0.0107 (10)

### Geometric parameters (Å, º)

**Table:**

O1—C16	1.312 (2)	C8—C9	1.362 (3)
O1—H1	0.89 (3)	C8—H8	0.9300
O2—C16	1.197 (2)	C9—C10	1.423 (3)
N1—C7	1.326 (2)	C9—C16	1.499 (3)
N1—C11	1.372 (2)	C10—C15	1.411 (3)
C1—C2	1.381 (3)	C10—C11	1.421 (3)
C1—C6	1.388 (3)	C11—C12	1.410 (3)
C1—C17	1.504 (3)	C12—C13	1.355 (3)
C2—C3	1.378 (3)	C12—H12	0.9300
C2—H2	0.9300	C13—C14	1.399 (3)
C3—C4	1.390 (3)	C13—H13	0.9300
C3—H3	0.9300	C14—C15	1.355 (3)
C4—C5	1.391 (3)	C14—H14	0.9300
C4—C7	1.480 (3)	C15—H15	0.9300
C5—C6	1.379 (3)	C17—H17A	0.9600
C5—H5	0.9300	C17—H17B	0.9600
C6—H6	0.9300	C17—H17C	0.9600
C7—C8	1.416 (3)		
			
C16—O1—H1	116.7 (16)	C10—C9—C16	123.30 (18)
C7—N1—C11	119.10 (15)	C15—C10—C11	118.45 (17)
C2—C1—C6	117.62 (18)	C15—C10—C9	124.72 (17)
C2—C1—C17	120.81 (19)	C11—C10—C9	116.82 (17)
C6—C1—C17	121.57 (19)	N1—C11—C12	118.12 (17)
C3—C2—C1	121.42 (18)	N1—C11—C10	122.65 (17)
C3—C2—H2	119.3	C12—C11—C10	119.23 (18)
C1—C2—H2	119.3	C13—C12—C11	120.2 (2)
C2—C3—C4	121.07 (18)	C13—C12—H12	119.9
C2—C3—H3	119.5	C11—C12—H12	119.9
C4—C3—H3	119.5	C12—C13—C14	120.9 (2)
C3—C4—C5	117.60 (18)	C12—C13—H13	119.5
C3—C4—C7	120.50 (16)	C14—C13—H13	119.5
C5—C4—C7	121.87 (17)	C15—C14—C13	120.5 (2)
C6—C5—C4	120.90 (19)	C15—C14—H14	119.8
C6—C5—H5	119.6	C13—C14—H14	119.8
C4—C5—H5	119.6	C14—C15—C10	120.7 (2)
C5—C6—C1	121.37 (18)	C14—C15—H15	119.6
C5—C6—H6	119.3	C10—C15—H15	119.6
C1—C6—H6	119.3	O2—C16—O1	124.26 (18)
N1—C7—C8	121.25 (18)	O2—C16—C9	122.20 (17)
N1—C7—C4	118.09 (16)	O1—C16—C9	113.53 (17)
C8—C7—C4	120.65 (17)	C1—C17—H17A	109.5
C9—C8—C7	121.01 (18)	C1—C17—H17B	109.5
C9—C8—H8	119.5	H17A—C17—H17B	109.5
C7—C8—H8	119.5	C1—C17—H17C	109.5
C8—C9—C10	119.15 (16)	H17A—C17—H17C	109.5
C8—C9—C16	117.55 (17)	H17B—C17—H17C	109.5
			
C6—C1—C2—C3	1.9 (3)	C16—C9—C10—C15	1.2 (3)
C17—C1—C2—C3	−178.5 (2)	C8—C9—C10—C11	0.2 (3)
C1—C2—C3—C4	−0.9 (3)	C16—C9—C10—C11	−179.99 (19)
C2—C3—C4—C5	−0.6 (3)	C7—N1—C11—C12	−178.7 (2)
C2—C3—C4—C7	177.28 (18)	C7—N1—C11—C10	1.6 (3)
C3—C4—C5—C6	1.1 (3)	C15—C10—C11—N1	177.7 (2)
C7—C4—C5—C6	−176.78 (19)	C9—C10—C11—N1	−1.2 (3)
C4—C5—C6—C1	−0.1 (3)	C15—C10—C11—C12	−2.0 (3)
C2—C1—C6—C5	−1.4 (3)	C9—C10—C11—C12	179.1 (2)
C17—C1—C6—C5	179.0 (2)	N1—C11—C12—C13	−179.1 (2)
C11—N1—C7—C8	−0.9 (3)	C10—C11—C12—C13	0.6 (4)
C11—N1—C7—C4	179.92 (17)	C11—C12—C13—C14	1.0 (4)
C3—C4—C7—N1	−25.0 (3)	C12—C13—C14—C15	−1.2 (4)
C5—C4—C7—N1	152.79 (19)	C13—C14—C15—C10	−0.3 (4)
C3—C4—C7—C8	155.78 (19)	C11—C10—C15—C14	1.9 (4)
C5—C4—C7—C8	−26.4 (3)	C9—C10—C15—C14	−179.3 (2)
N1—C7—C8—C9	−0.1 (3)	C8—C9—C16—O2	44.3 (3)
C4—C7—C8—C9	179.09 (18)	C10—C9—C16—O2	−135.5 (2)
C7—C8—C9—C10	0.4 (3)	C8—C9—C16—O1	−134.5 (2)
C7—C8—C9—C16	−179.40 (18)	C10—C9—C16—O1	45.7 (3)
C8—C9—C10—C15	−178.6 (2)		

### Hydrogen-bond geometry (Å, º)

**Table:**

*D*—H···*A*	*D*—H	H···*A*	*D*···*A*	*D*—H···*A*
O1—H1···N1^i^	0.89 (3)	1.89 (3)	2.763 (2)	168 (2)
C3—H3···O1^ii^	0.93	2.51	3.233 (2)	135

Symmetry codes: (i) −*x*+1, *y*−1/2, −*z*+1/2; (ii) −*x*, *y*+1/2, −*z*+1/2.

## References

[bb1] Blackburn, A. C., Dobson, A. J. & Gerkin, R. E. (1996). *Acta Cryst.* C**52**, 409–411.

[bb2] Clark, R. C. & Reid, J. S. (1995). *Acta Cryst.* A**51**, 887–897.

[bb3] Cremer, D. & Pople, J. A. (1975). *J. Am. Chem. Soc.* **97**, 1354–1358.

[bb4] Deady, L. W., Desneves, J., Kaye, A. J., Finlay, G. J., Bagvley, B. C. & Denny, W. A. (2011). *Bioorg. Med. Chem.* **9**, 445–452. 10.1016/s0968-0896(00)00264-9 11249136

[bb5] Deady, L. W., Desneves, J., Kaye, A. J., Thompson, M., Finlay, G. L., Bagvley, B. C. & Denny, W. A. (1999). *Bioorg. Med. Chem.* **7**, 2801–2809. 10.1016/s0968-0896(99)00231-x 10658584

[bb6] Dolomanov, O. V., Bourhis, L. J., Gildea, R. J., Howard, J. A. K. & Puschmann, H. (2009). *J. Appl. Cryst.* **42**, 339–341.

[bb7] Kalluraya, B. & Sreenivasa, S. (1998). *Il Farmaco*, **53**, 399–404. 10.1016/s0014-827x(98)00037-8 9764472

[bb8] Kravchenko, D., Kazakova, Y. A., Kysil, M., Tkachenko, S. E., Malarchuk, S., Okun, I. M., Balakin, K. V. & Ivachtchenko, A. V. (2005). *J. Med. Chem.* **48**, 3680–3683. 10.1021/jm048987t 15916416

[bb9] Oxford Diffraction (2009). *CrysAlis PRO* Oxford Diffraction Ltd, Yarnton, England.

[bb10] Pfitzinger, W. (1886). *J. Prakt. Chem.* **33**, 100.

[bb11] Sheldrick, G. M. (2008). *Acta Cryst.* A**64**, 112–122. 10.1107/S0108767307043930 18156677

[bb12] Tseng, C.-H., Chen, Y.-L., Lu, P.-J., ang, C.-N. & Tzeng, C.-C. (2008). *Bioorg. Med. Chem.* **16**, 3153–3161. 10.1016/j.bmc.2007.12.028 18180162

